# How are guidelines on allergic respiratory diseases implemented in primary care in Canada?

**DOI:** 10.1186/1710-1492-6-S4-A9

**Published:** 2010-12-10

**Authors:** Louis-Philippe Boulet

**Affiliations:** 1 Institut universitaire de cardiologie et de pneumologie de Québec, Université Laval, Québec, G1V 4G5, Canada

## 

Asthma is still not optimally controlled in Canada, as in other countries [1,2]. As an effort to determine what could be optimal care of this chronic condition, Clinical Practice Guidelines (CPGs) have been produced and revised regularly in the last two decades, although recent reports suggest that they are not still sufficiently implemented, particularly into primary care [1-4]. Cabana *et al*. had reported, following a systematic literature review of studies on barriers to adherence to CPGs, that more than half of physicians were not aware of the exact content of current guidelines; common barriers to guidelines implementation included a lack of agreement and/or motivation to change, a lack of self-efficacy, inertia of previous practice, and external factors, such as lack of time and insufficient resources [4].

In regard to asthma, we previously reported data from questionnaires completed by 2,605 Canadian practitioners [5]; we observed that Canadian Asthma Consensus Guidelines (CACG) were considered well known only by a minority of Primary care Physicians (PCP) and that significant discrepancies existed between this Guideline’s recommendations and current care, such as a low use of objective measures of airflow limitation to assist with diagnosis, difficulties with proper assessment of medication needs, a low provision of written action plans for patients and an infrequent referral to other health professionals for asthma education. In keeping with this initial study, following a recent Canadian survey, we proposed that an insufficient use of objective measures for the diagnosis of asthma explained the over-diagnosis of asthma observed [6].

More recently, using a sample of patients treated in primary care in Canada, we confirmed that many patients still do not enjoy adequate guideline-defined asthma control, particularly women, current smokers, older patients and those who lack a written action plan for exacerbation management [1]. However, primary care physicians recognized a lack of control among most asthma patients and were likely to recommend appropriate medication changes and aftercare to patients who fail to achieve guideline targets for asthma control. This, therefore, suggests that although there have been improvements in guideline implementation, some care gaps still persist.

In order to better understand how Canadian guidelines are implemented and to develop targeted strategies to improve their translation into current care, we developed a new tool, the *Physicians’ Practice Assessment Questionnaire* (PPAQ) [7]. As an initial assessment of this tool, implementation of fourteen key-recommendations on asthma management from the most recent CACG was evaluated in a group of 47 general practitioners^7^. Validation of the questionnaire showed good internal consistency and good test-retest, and revealed that the lowest self-reported rates of implementation were for: 1) referral for patient education; 2) provision of a written action plan; 3) regular assessment of inhaler technique; and 4) referral to a specialist for difficult to control asthma or uncertain diagnosis. This suggests that in order to improve asthma control, these specific behaviors should be addressed and translation of these recommendations facilitated.

Finally, in regard to rhinitis, under-diagnosis and under-treatment of rhinitis seems common and although international guidelines are available, we need to better understand how physicians integrate their recommendations into care [8]. Barriers and facilitators of guidelines’ translation into current care should be further addressed.
